# Crystal structure and Hirshfeld surface analysis of (*E*)-1-{2,2-di­bromo-1-[4-(*tert*-but­yl)phen­yl]ethen­yl}-2-(3,4-di­methyl­phen­yl)diazene

**DOI:** 10.1107/S2056989025005390

**Published:** 2025-07-01

**Authors:** Namig G. Shikhaliyev, Jonathan Cisterna, Ayten M. Gajar, Ayten A. Niyazova, Ali N. Khalilov, Victor N. Khrustalev, Alebel N. Belay, Abel M. Maharramov

**Affiliations:** a Department of Chemical Engineering, Baku Engineering University, Hasan Aliyev Str. 120, AZ 0101 Baku, Azerbaijan; b Departamento de Química, Universidad Católica del Norte, Av. Angamos 0610, Antofagasta, Chile; c Department of Chemistry, Baku State University, Z. Khalilov str. 23, Az 1148, Baku, Azerbaijan; d "Composite Materials" Scientific Research Center, Azerbaijan State Economic University (UNEC), Murtuza Mukhtarov str. 194, Az 1065, Baku, Azerbaijan; e Peoples’ Friendship University of Russia (RUDN University) Miklukho-Maklay St 6 Moscow 117198 Russian Federation; f N. D. Zelinsky Institute of Organic, Chemistry RAS, Leninsky Prosp. 47, Moscow, 119991, Russian Federation; g Department of Chemistry, Bahir Dar University, PO Box 79, Bahir Dar, Ethiopia; University of Missouri-Columbia, USA

**Keywords:** azo compounds, Hirshfeld surface analysis, supra­molecular inter­actions, crystal structure

## Abstract

The title azo compound, C_20_H_22_Br_2_N_2_, crystallizes in the triclinic space group *P*1 (No. 2) with two independent mol­ecules in the asymmetric unit. The mol­ecular structure was analyzed using spectroscopic and crystallographic techniques, confirming the expected configuration and electronic environment. Hirshfeld surface analysis revealed key non-covalent inter­actions such as C—H⋯π, C—H⋯Br, π–π stacking, and halogen bonding, which consolidate the crystal structure. The study provides valuable insights into the structural features and inter­molecular inter­actions of this brominated azo compound, which may have potential applications in optoelectronic materials, dyes, and mol­ecular switches.

## Abstract 2

The title azo compound crystallizes in the triclinic space group *P*1 with two independent mol­ecules in the asymmetric unit. Hirshfeld surface analysis and energy framework calculations revealed key non-covalent inter­actions such as C—H⋯π, C—H⋯Br, π–π stacking, and halogen bonding, which consolidate the crystal structure.

## 1. Chemical context

Azo compounds represent a significant class of organic mol­ecules characterized by a diazenyl (–N=N–) functional group linking two aromatic systems (Shikhaliyev *et al.*, 2018 ▸; Naghiyev *et al.*, 2019 ▸). These compounds have garnered considerable inter­est due to their diverse applications in dyes, pigments, pharmaceuticals, and advanced materials, including liquid crystals and organic semiconductors (Shixaliyev *et al.*, 2013 ▸; Shikhaliyev *et al.*, 2019 ▸; Tahirli *et al.*, 2024 ▸). The introduction of various substituents on the aromatic rings can fine-tune their electronic, optical, and chemical properties, making them highly versatile for industrial and scientific applications (Gurbanov *et al.*, 2021 ▸; Mahmoudi *et al.*, 2021 ▸).

The structure under investigation features a di­bromo­vinyl­idenebenzyl moiety and a methyl-substituted phenyl group linked *via* an azo (–N=N–) bridge (Israyilova *et al.*, 2016 ▸; Çelikesir *et al.*, 2022 ▸). The di­bromo­alkene moiety enhances the reactivity, allowing for further functionalization and potential applications in cross-coupling reactions, while alkyl substituents on the phenyl ring contribute to improved solubility and stability (Dobrounig *et al.*, 2017 ▸; Atioğlu *et al.*, 2022*a* ▸,*b* ▸; Khalilov *et al.*, 2022 ▸). Although the *tert*-butyl­phenyl ring is electronically decoupled due to the lack of conjugation with the azo and vinyl units, the compound still contains a conjugated π-system involving the diazo group and the *p*-methyl­phenyl ring. This partial conjugation may contribute to inter­esting photophysical and electronic properties, which are relevant for potential applications in azo dyes, mol­ecular switches, and optoelectronic materials., azo dyes, and mol­ecular switches (Akkurt *et al.*, 2022 ▸; Shikhaliyev *et al.*, 2021 ▸).

While synthetic azo dyes have been widely studied and commercially utilized, naturally occurring dyes containing the azo (–N=N–) functional group are infrequent (Özkaraca *et al.*, 2020**a* ▸,b* ▸). Most natural dyes are based on anthra­quinone, flavonoid, or indigoid chromophores. Despite their limited natural occurrence, azo compounds are extensively synthesized because of their excellent color stability, tunability, and strong chromophoric properties (Magerramov *et al.*, 2018 ▸; Benkhaya *et al.*, 2020 ▸).
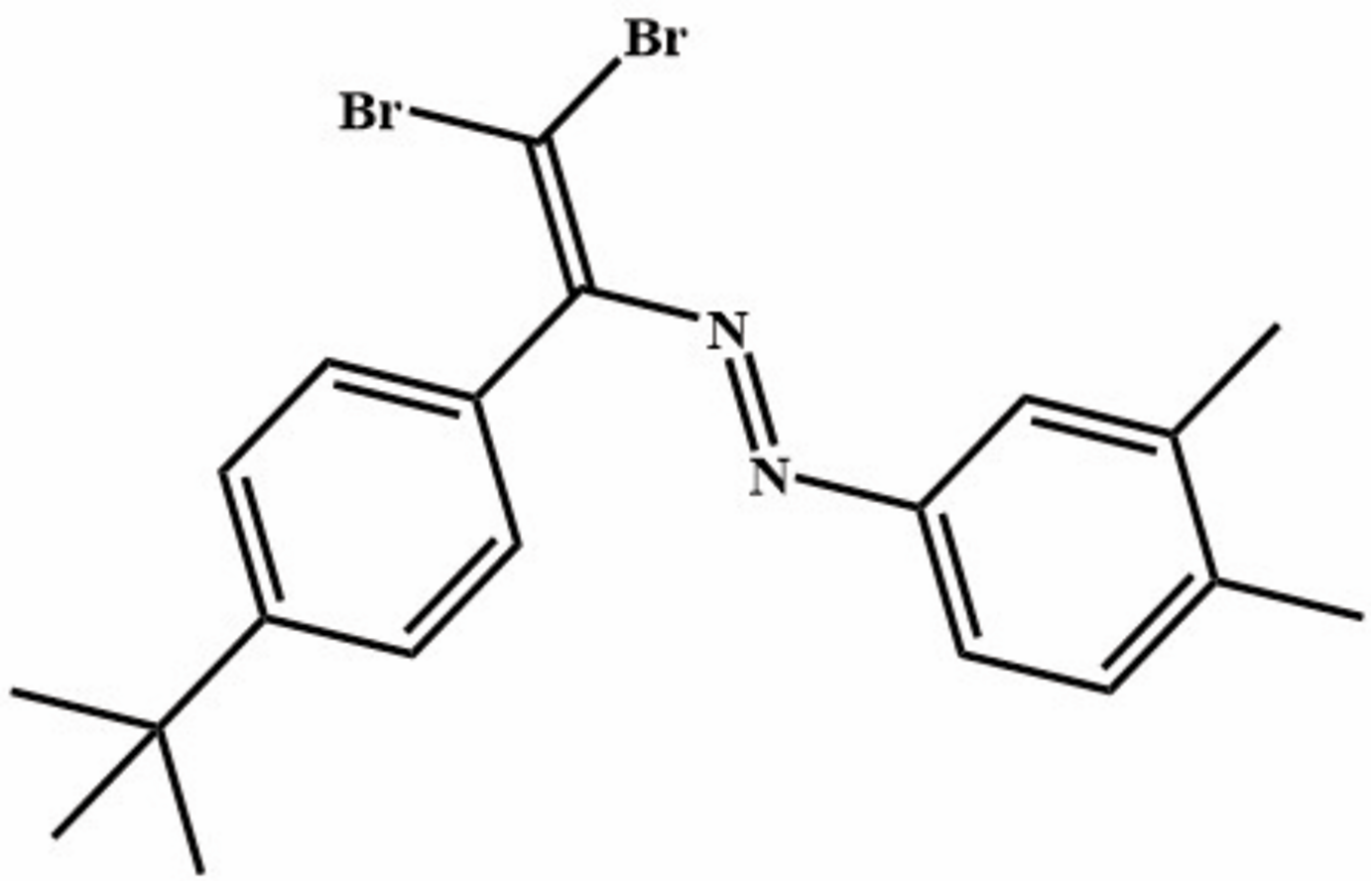


Given the significance of azo compounds in material science and organic synthesis, the present study reports the synthesis and crystal structure of (*E*)-1-{2,2-di­bromo-1-[4-(*tert*-but­yl)phen­yl]ethen­yl}-2-(3,4-di­methyl­phen­yl)diazene in which the di­bromo­vinyl and *tert*-butyl groups were incorporated to modulate the azo scaffold’s reactivity and solubility profiles.

## 2. Structural commentary

**Figure 1:**
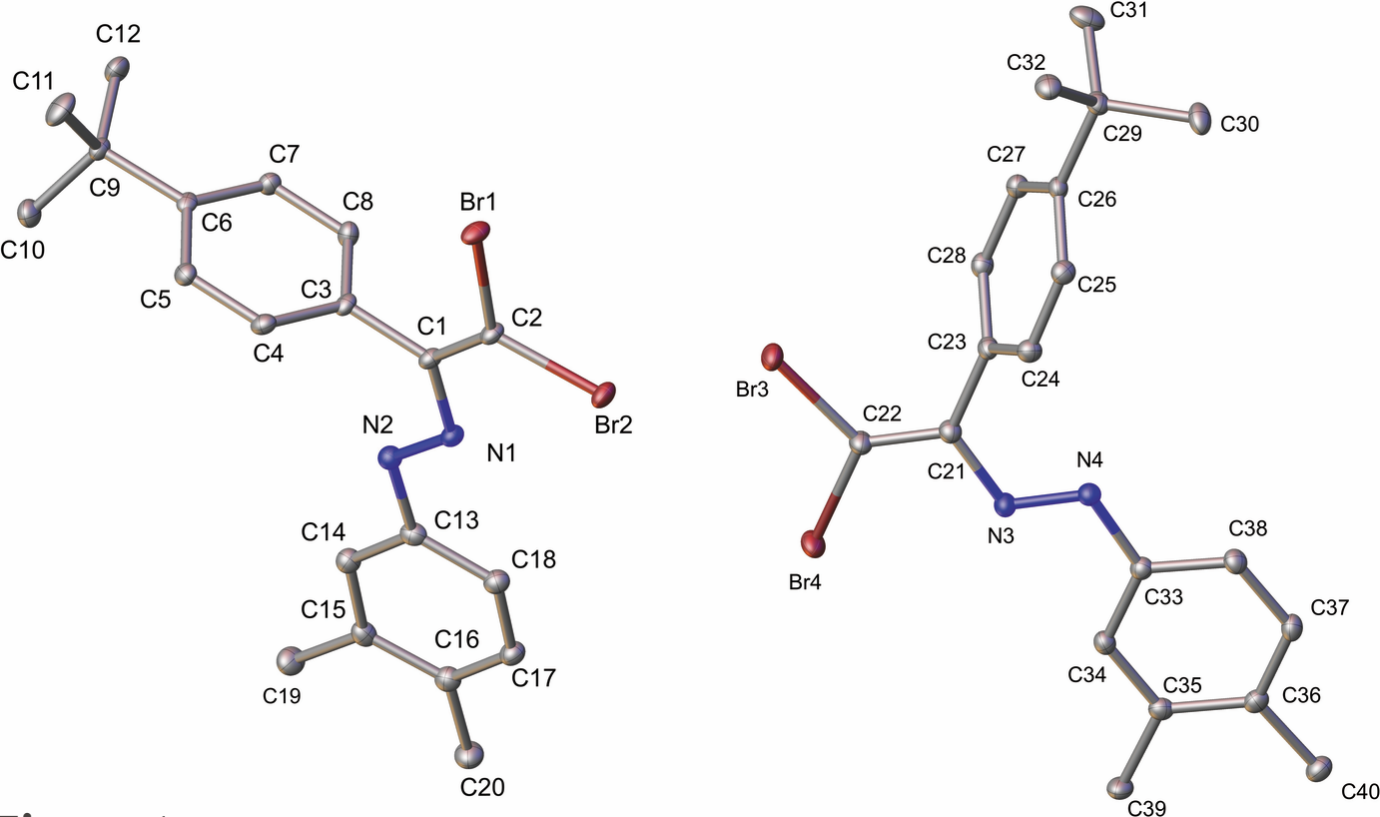
Ellipsoid plots (30% probability) of fragments 1(left) and 2 (right). Hydrogen atoms are omitted for the sake of clarity.

The title compound crystallizes in the triclinic cell system with space group *P*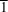 (No. 2), with two mol­ecules in the asymmetric unit (*Z* = 4). The mol­ecular structure of the compound agrees with the spectroscopic characterization and the proposed structure, in a centrosymmetric setting, with normal bond distances (Allen *et al.*, 1987 ▸) and angles, being the *E*-isomer confirmed as mol­ecular structure within the crystal (see Fig. 1 ▸). The independent mol­ecules are labeled fragment 1 and 2, correlative and sorted atom labels, The diazo bond environments in each mol­ecule are practically coplanar, apart from the 4-*tert-*butyl­phenyl moiety in both cases. There is a lack of conjugation between the *tert*-butyl­phenyl group and the adjacent vinyl group, with dihedral angles of 89.2 (2) and 66.8 (2)° for fragments 1 and 2, respectively. The dihedral angles between these two fragments indicate that in both cases the aromatic ring is twisted out of planarity relative to the vinyl moiety, effectively insulating it electronically from the azo group. This lack of conjugation may have important implications for the electronic distribution and potential reactivity of the mol­ecule.

The origin of this non-conjugated geometry warrants further consideration. One possible explanation is steric hindrance introduced by the bulky *tert*-butyl substituent, which may prevent the coplanarity required for extended π-conjugation. Alternatively, this conformation could be stabilized or enforced by crystal-packing effects, as inter­molecular forces may favor a geometry that minimizes unfavorable contacts or facilitates specific packing motifs, such as herringbone arrangements.

## 3. Supra­molecular features

**Figure 2:**
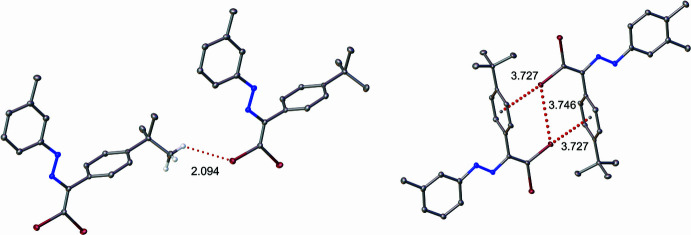
C—H⋯Br interactions and halogen bonds found in the crystal structure of the title compound.

**Figure 3:**
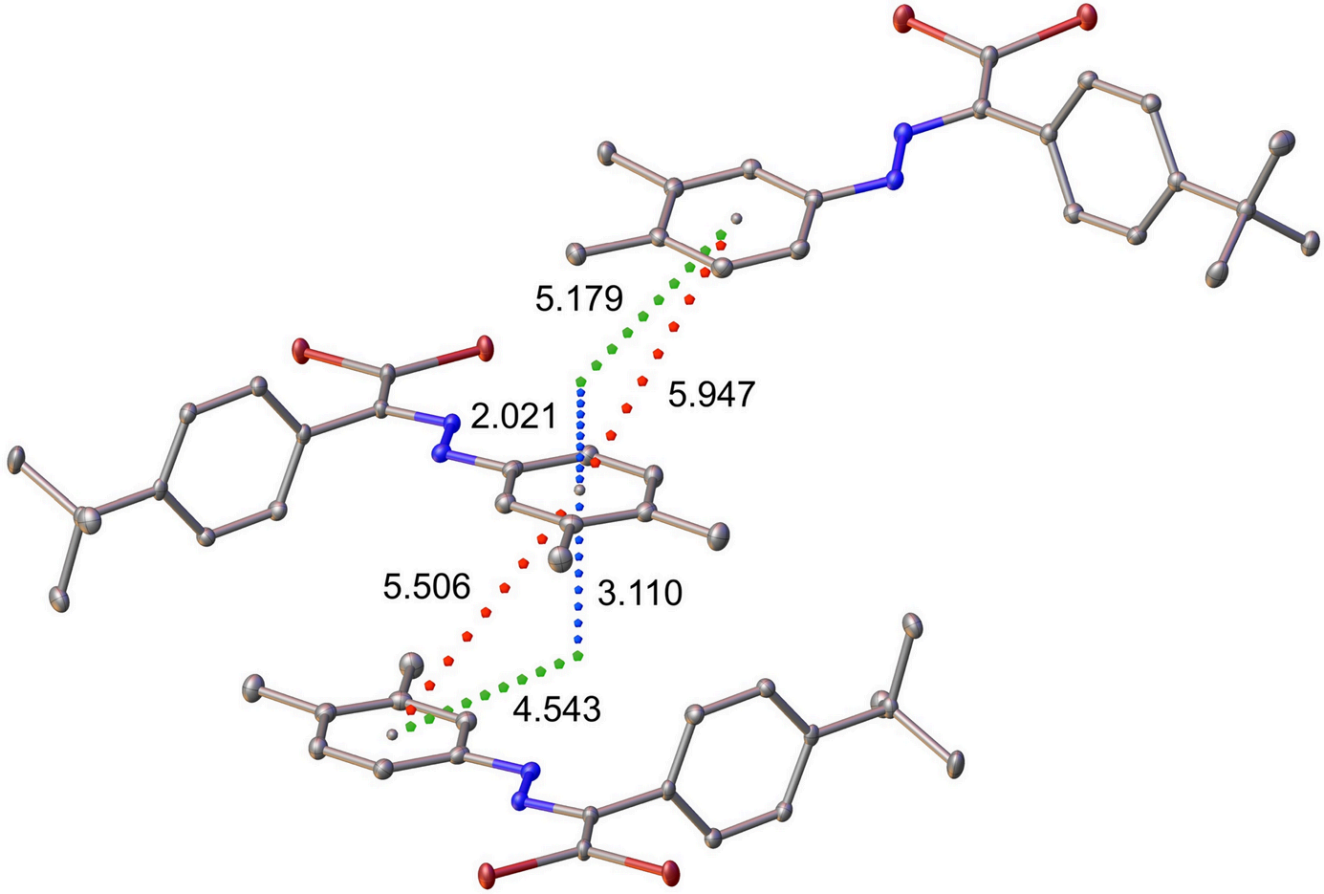
π–π-like stacking in the title compound.

The crystal packing of the title compound does not exhibit geometrical parameters consistent with classical hydrogen bonds (Steiner, 2002 ▸). Instead, the structure is consolidated by inter­molecular inter­actions such as H⋯π, C—H⋯Br, halogen bonds, and parallel-displaced π–π stacking (see Fig. 2 ▸). The π–π inter­actions exceed conventional parameters for this type of inter­action (Carter-Fenk & Herbert, 2020 ▸). The centroid–centroid distances are 5.5060 (14) and 5.9474 (13) Å (red dotted lines), with perpendicular ring plane separations of 3.1104 (9) and 2.2010 (9) Å (blue dotted lines). The slippage values are 4.543 and 5.060 Å (green dotted lines), observed between the C13–C18 ring and its symmetry equivalent at 1 − *x*, 1 − *y*, 2 − *z*) and between the C13–C18 and C33–C38(*x*, 2 − *y*, 1 − *z*) rings, respectively (Fig. 3 ▸).

It is worth noting that a *Z*′ value of 2 for two conformationally identical mol­ecules is uncommon. This appears to arise from the fact that, although the mol­ecules adopt a herringbone packing *motif*, they cannot do so without a relative offset due to the dihedral angle. This observation suggests that the mol­ecular conformation is particularly stable.

**Figure 4:**
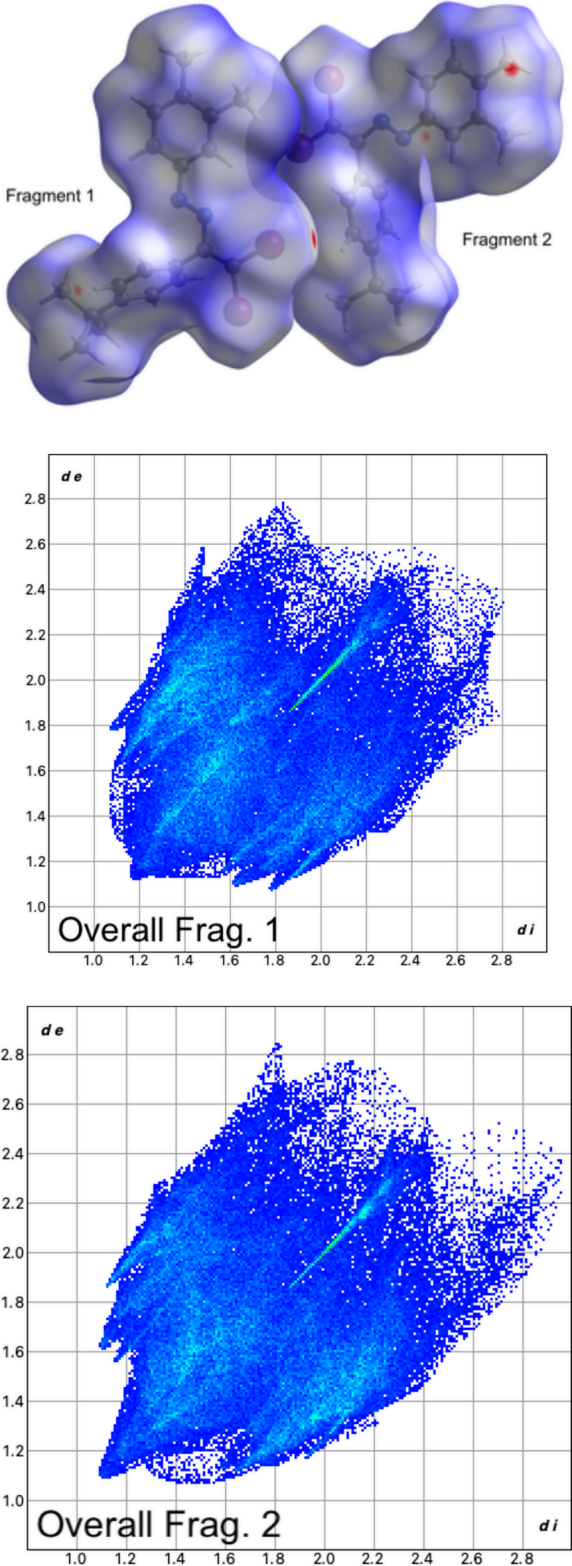
Overall Hirshfeld surface for fragments 1 and 2 and their respective overall fingerprint plots.

Hirshfeld surface analysis was conducted for each fragment individually to gain a deeper understanding of all contributions. These inter­actions are visualized as red (*d*_norm_ < vdW radii), white (*d_norm_* = vdW radii), and blue (*d*_norm_ > vdW radii) spots on the *d*_norm_ surfaces for all compounds along with fingerprint plots mapped with dnorm (where *d*_norm_*= d*_i_*+ d*_e_) (Fig. 4 ▸). H⋯Br contacts are observed to contribute around 20% with *d*_i_*+ d*_e_ ≃ 2.1 Å, generating a dimeric setting between the two fragments within the crystal structure. This is observed in similar compounds with a similar behavior without the formation of supra­molecular aggregates in the crystal structure (Maharramov *et al.* 2019 ▸). H⋯π contacts contribute around 20% with *d*_i_*+ d*_e_ ≃ 3.0 Å, generating the stacking between the fragments along the *a*-axis direction. Although weaker than conventional hydrogen bonds, these inter­actions can play a crucial role in crystal packing, mol­ecular recognition, and other supra­molecular processes (Nagarajan *et al.* 2014 ▸; Rajagopal *et al.* 2016 ▸). H⋯H contacts and π–π contacts make negligible contributions. Br⋯Br contacts are observed with a contribution of around 3.5%, and *d*_i_*+ d*_e_ ≃ 3.7 and 4.5 Å, generating a tetra­mer in the crystal structure along the *b*-axis direction. These inter­actions, although often weak, are recognized for their capacity to influence the overall packing and consolidation of mol­ecular crystals (Marek *et al.* 2018 ▸; Cavallo *et al.* 2016 ▸; Varadwaj *et al.* 2019 ▸).

It is also worth noting that the presence of electron-donating substituents on the mol­ecule can modulate the electron-density distribution, potentially altering the nature and strength of inter­actions involving bromine atoms. This suggests that substituent effects may play a role not only in intra­molecular properties, but also in dictating the inter­molecular landscape of the crystal packing.

## 4. Database survey

A Cambridge Structural Database (CSD; 2022.3 Version, November 2024 update; Groom *et al.*, 2016 ▸) search for the (*E*)-1-{2,2-di­bromo-1-[4-(*tert*-but­yl)phen­yl]vin­yl}-2-(3,4-di­methyl­phen­yl)diazene unit yielded 90 hits. Refining the search to include structures containing Br atoms gave just four compounds closely resembling the title compound, *viz*. ECUDAL [(I); Atioğlu *et al.*, 2022*b* ▸], HEHKEO [(II); Akkurt *et al.*, 2022 ▸], PAXDOL [(III); Çelikesir *et al.*, 2022 ▸], and TAZDIL [(IV); Atioğlu *et al.*, 2022*a* ▸].

In the crystal structure of (I), the mol­ecules form inversion dimers *via* short halogen–halogen contacts [Br3⋯Br3 = 4.0103 (6) Å, C12—Br3⋯Br3 = 72.23 (6)°], which are shorter than the van der Waals radius sum of 5.55 Å for two bromine atoms. Additional directional inter­actions include Br1⋯O2 contacts [3.137 (19) Å] along the [010] direction and π–π stacking inter­actions between aromatic rings are also present, with centroid–centroid distances of 3.7305 (11) Å [perpendicular ring plane separations of 3.3964 (8) and 3.1119 (8) Å, and slippage values of 2.057 and 1.543 Å].

In (II), the structure features the same halogen contacts as in (I), but additionally presents π⋯Br contacts with a Br1–4-nitro mono-substituted ring distance of 3.546 (17) Å. Unlike in compound (I), no π–π stacking inter­actions are observed in this structure.

The crystal structure of (III) exhibits inter­molecular hydrogen-bonding inter­actions between the aromatic rings and the oxygen of the nitro moiety [*D*⋯H = 2.51 Å, *D*⋯*A* = 3.3244 (18) Å, *D*—H⋯*A* =144°. Br⋯O contacts (2.983 Å) are also present.

Finally in (IV), several hydrogen-bonding inter­actions consolidate the crystal structure due to the inversion center present in the unit cell. π–π stacking inter­actions are found, but at larger distances than conventional values (weighted distance = 4.079 Å). The structure also exhibits halogen–π inter­actions, with a mean distance of 3.503 Å.

## 5. Synthesis and crystallization

The title compound was obtained using a previously reported procedure (Shikhaliyev *et al.*, 2018 ▸), with appropriate modifications for the brominated analog. A 20 ml screw-neck vial was charged with DMSO (10 mL), (*E*)-1-[4-(*tert*-but­yl)benzyl­idene]-2-(3,4-di­methyl­phen­yl)hydrazine (281 mg, 1 mmol), tetra­methyl­ethylenedi­amine (TMEDA) (295 mg, 2.5 mmol), CuCl (2 mg, 0.02 mmol) and CBr_4_ (3 mmol). After 1–3 h (until TLC analysis showed complete consumption of corresponding Schiff base), the reaction mixture was poured into an 0.01 *M* solution of HCl (100 ml, pH = 2), and extracted with di­chloro­methane (3 × 20 ml). The combined organic phase was washed with water (3 × 50 ml) and brine (30 ml), dried over anhydrous Na_2_SO_4_ and concentrated *in vacuo*. The residue was purified by column chromatography on silica gel using appropriate mixtures of hexane and di­chloro­methane (3:1, *v*/*v*). The resulting title compound was obtained as an orange crystalline solid, m.p. 388 K; yield: 34%.

^1^H NMR (300 MHz, CDCl_3_) δ 7.69 (*s*, 1H), 7.67 (*d*, *J* = 8 Hz, 1H), 7.54 (*d*, *J* = 8.4 Hz, 2H), 7.32 (*d*, *J* = 8 Hz, 1H),7.21 (*d*, *J* = 8.4 Hz, 2H), 2.33 (*s*, 6H), 1.44 (*s*, 9H). ^13^C NMR (75 MHz, CDCl3) δ 153.7, 152.3, 151.9, 142.6, 138.5, 134.6, 132.5, 127.9, 127.7, 125.7, 123.8, 121.5, 34.5, 31.9, 20.7, 19.4.

## 6. Refinement

Crystal data, data collection and structure refinement details are summarized in Table 1 ▸. Hydrogen atoms were found in difference-Fourier maps, and included in the refinement using riding models, with constrained distances set to 0.95 Å (C*sp*^2^—H) and 0.98 Å (*R*CH_3_). *U*_iso_(H) parameters were set to values of either 1.2*U*_eq_ or 1.5*U*_eq_ (*R*CH_3_ only) of the attached atom.

## Supplementary Material

Crystal structure: contains datablock(s) I. DOI: 10.1107/S2056989025005390/ev2015sup1.cif

Structure factors: contains datablock(s) I. DOI: 10.1107/S2056989025005390/ev2015Isup4.hkl

Supporting information file. DOI: 10.1107/S2056989025005390/ev2015Isup3.cml

CCDC reference: 2465012

Additional supporting information:  crystallographic information; 3D view; checkCIF report

## Figures and Tables

**Table 1:** Experimental details

Crystal data	
Chemical formula	C_20_H_22_Br_2_N_2_
*M*_r_	450.20
Crystal system, space group	Triclinic, *P* 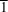
Temperature (K)	100
*a*, *b*, *c* (Å)	9.89357 (5), 12.23384 (7), 17.12566 (9)
α, β, γ (°)	76.6038 (5), 89.3055 (4), 73.3449 (5)
*V* (Å^3^)	1928.56 (2)
*Z*	4
Radiation type	Cu *K*α
μ (mm^−1^)	5.34
Crystal size (mm)	0.31 × 0.25 × 0.21

Data collection	
Diffractometer	XtaLAB Synergy, Dualflex, HyPix
Absorption correction	Multi-scan (*CrysAlis PRO*; Rigaku OD, 2021 ▸)
*T*_min_, *T*_max_	0.504, 1.000
No. of measured, independent and observed [*I* > 2σ(*I*)] reflections	59739, 8330, 7876
*R*_int_	0.049
(sin θ/λ)_max_ (Å^−1^)	0.638

Refinement	
*R*[*F*^2^ > 2σ(*F*^2^)], *wR*(*F*^2^), *S*	0.035, 0.098, 1.09
No. of reflections	8330
No. of parameters	443
H-atom treatment	H-atom parameters constrained
Δρ_max_, Δρ_min_ (e Å^−3^)	1.01, −0.86

Computer programs: *CrysAlis PRO* (Rigaku OD, 2021 ▸), *SHELXT* (Sheldrick, 2015*a* ▸), *SHELXL* (Sheldrick, 2015*b* ▸) and *SHELXTL* (Sheldrick, 2008 ▸).

## supplementary crystallographic information

### (E)-1-{2,2-Dibromo-1-[4-(tert-butyl)phenyl]ethenyl}-\ 2-(3,4-dimethylphenyl)diazene Crystal data

**Table:**

C_20_H_22_Br_2_N_2_	*Z* = 4
*M**_r_* = 450.20	*F*(000) = 904
Triclinic, *P*1	*D*_x_ = 1.551 Mg m^−^^3^
*a* = 9.89357 (5) Å	Cu *K*α radiation, λ = 1.54184 Å
*b* = 12.23384 (7) Å	Cell parameters from 36360 reflections
*c* = 17.12566 (9) Å	θ = 2.7–79.4°
α = 76.6038 (5)°	µ = 5.34 mm^−^^1^
β = 89.3055 (4)°	*T* = 100 K
γ = 73.3449 (5)°	Prism, red
*V* = 1928.56 (2) Å^3^	0.31 × 0.25 × 0.21 mm

### (E)-1-{2,2-Dibromo-1-[4-(tert-butyl)phenyl]ethenyl}-\ 2-(3,4-dimethylphenyl)diazene Data collection

**Table:**

XtaLAB Synergy, Dualflex, HyPix diffractometer	8330 independent reflections
Radiation source: micro-focus sealed X-ray tube	7876 reflections with *I* > 2σ(*I*)
Detector resolution: 0.78 pixels mm^-1^	*R*_int_ = 0.049
φ and ω scans	θ_max_ = 79.6°, θ_min_ = 2.7°
Absorption correction: multi-scan (CrysAlisPro; Rigaku OD, 2021)	*h* = −11→12
*T*_min_ = 0.504, *T*_max_ = 1.000	*k* = −15→15
59739 measured reflections	*l* = −21→21

### (E)-1-{2,2-Dibromo-1-[4-(tert-butyl)phenyl]ethenyl}-\ 2-(3,4-dimethylphenyl)diazene Refinement

**Table:**

Refinement on *F*^2^	Primary atom site location: difference Fourier map
Least-squares matrix: full	Secondary atom site location: difference Fourier map
*R*[*F*^2^ > 2σ(*F*^2^)] = 0.035	Hydrogen site location: inferred from neighbouring sites
*wR*(*F*^2^) = 0.098	H-atom parameters constrained
*S* = 1.09	*w* = 1/[σ^2^(*F*_o_^2^) + (0.0523*P*)^2^ + 1.7466*P*] where *P* = (*F*_o_^2^ + 2*F*_c_^2^)/3
8330 reflections	(Δ/σ)_max_ = 0.001
443 parameters	Δρ_max_ = 1.01 e Å^−^^3^
0 restraints	Δρ_min_ = −0.86 e Å^−^^3^

### (E)-1-{2,2-Dibromo-1-[4-(tert-butyl)phenyl]ethenyl}-\ 2-(3,4-dimethylphenyl)diazene Special details

**Table:**

Experimental. CrysAlisPro 1.171.41.117a (Rigaku Oxford Diffraction, 2021) Empirical absorption correction using spherical harmonics, implemented in SCALE3 ABSPACK scaling algorithm.
Geometry. All esds (except the esd in the dihedral angle between two l.s. planes) are estimated using the full covariance matrix. The cell esds are taken into account individually in the estimation of esds in distances, angles and torsion angles; correlations between esds in cell parameters are only used when they are defined by crystal symmetry. An approximate (isotropic) treatment of cell esds is used for estimating esds involving l.s. planes.

### (E)-1-{2,2-Dibromo-1-[4-(tert-butyl)phenyl]ethenyl}-\ 2-(3,4-dimethylphenyl)diazene Fractional atomic coordinates and isotropic or equivalent isotropic displacement parameters (Å^2^)

**Table:**

	*x*	*y*	*z*	*U*_iso_*/*U*_eq_	
Br1	0.39020 (3)	0.65399 (2)	0.49638 (2)	0.02673 (8)	
Br2	0.34648 (3)	0.87116 (2)	0.57292 (2)	0.02590 (8)	
N1	0.35259 (18)	0.69310 (16)	0.72925 (11)	0.0193 (3)	
N2	0.34989 (19)	0.62342 (17)	0.79595 (11)	0.0209 (3)	
C1	0.3699 (2)	0.63915 (18)	0.66366 (12)	0.0189 (4)	
C2	0.3679 (2)	0.70933 (19)	0.59018 (13)	0.0210 (4)	
C3	0.3979 (2)	0.50950 (18)	0.67648 (12)	0.0188 (4)	
C4	0.2892 (2)	0.4581 (2)	0.67752 (13)	0.0216 (4)	
H4	0.1939	0.5062	0.6681	0.026*	
C5	0.3195 (2)	0.33693 (19)	0.69232 (13)	0.0208 (4)	
H5	0.2441	0.3030	0.6934	0.025*	
C6	0.4588 (2)	0.26337 (18)	0.70571 (11)	0.0177 (4)	
C7	0.5661 (2)	0.31621 (19)	0.70445 (13)	0.0201 (4)	
H7	0.6616	0.2685	0.7134	0.024*	
C8	0.5364 (2)	0.43807 (19)	0.69034 (13)	0.0222 (4)	
H8	0.6114	0.4722	0.6902	0.027*	
C9	0.4882 (2)	0.12987 (18)	0.72170 (12)	0.0200 (4)	
C10	0.4240 (3)	0.0863 (2)	0.80055 (15)	0.0304 (5)	
H10A	0.4445	0.0007	0.8118	0.046*	
H10B	0.3215	0.1227	0.7957	0.046*	
H10C	0.4651	0.1076	0.8446	0.046*	
C11	0.4221 (3)	0.0985 (2)	0.65293 (17)	0.0360 (6)	
H11A	0.4594	0.1299	0.6021	0.054*	
H11B	0.3193	0.1326	0.6502	0.054*	
H11C	0.4453	0.0129	0.6624	0.054*	
C12	0.6470 (3)	0.0659 (2)	0.72932 (16)	0.0295 (5)	
H12A	0.6912	0.0835	0.7739	0.044*	
H12B	0.6898	0.0923	0.6792	0.044*	
H12C	0.6614	−0.0190	0.7397	0.044*	
C13	0.3361 (2)	0.6775 (2)	0.86203 (13)	0.0221 (4)	
C14	0.3347 (2)	0.6052 (2)	0.93741 (13)	0.0235 (4)	
H14	0.3405	0.5252	0.9416	0.028*	
C15	0.3249 (2)	0.6471 (2)	1.00761 (14)	0.0240 (4)	
C16	0.3187 (2)	0.7646 (2)	1.00061 (14)	0.0247 (4)	
C17	0.3173 (2)	0.8378 (2)	0.92485 (14)	0.0250 (4)	
H17	0.3102	0.9181	0.9205	0.030*	
C18	0.3259 (2)	0.7964 (2)	0.85567 (14)	0.0237 (4)	
H18	0.3250	0.8476	0.8045	0.028*	
C19	0.3217 (3)	0.5654 (2)	1.08795 (14)	0.0311 (5)	
H19A	0.3261	0.4875	1.0802	0.047*	
H19B	0.2341	0.5964	1.1132	0.047*	
H19C	0.4030	0.5590	1.1227	0.047*	
C20	0.3146 (3)	0.8120 (2)	1.07465 (15)	0.0323 (5)	
H20A	0.3183	0.8934	1.0592	0.048*	
H20B	0.3958	0.7640	1.1116	0.048*	
H20C	0.2270	0.8095	1.1013	0.048*	
Br3	0.24367 (3)	0.15502 (2)	0.41709 (2)	0.02955 (8)	
Br4	0.19508 (3)	0.36576 (2)	0.50063 (2)	0.03296 (8)	
N3	0.1094 (2)	0.51443 (17)	0.33545 (11)	0.0220 (4)	
N4	0.07769 (19)	0.58727 (16)	0.26830 (11)	0.0204 (3)	
C21	0.1507 (2)	0.39520 (19)	0.33196 (13)	0.0214 (4)	
C22	0.1889 (2)	0.3189 (2)	0.40421 (13)	0.0241 (4)	
C23	0.1561 (2)	0.35732 (18)	0.25522 (13)	0.0206 (4)	
C24	0.0330 (2)	0.3772 (2)	0.20823 (14)	0.0233 (4)	
H24	−0.0560	0.4162	0.2250	0.028*	
C25	0.0405 (2)	0.3402 (2)	0.13751 (13)	0.0227 (4)	
H25	−0.0442	0.3538	0.1065	0.027*	
C26	0.1699 (2)	0.28308 (18)	0.11024 (12)	0.0194 (4)	
C27	0.2918 (2)	0.26499 (19)	0.15739 (13)	0.0213 (4)	
H27	0.3811	0.2274	0.1403	0.026*	
C28	0.2851 (2)	0.30098 (19)	0.22908 (13)	0.0219 (4)	
H28	0.3696	0.2869	0.2604	0.026*	
C29	0.1723 (2)	0.2451 (2)	0.03092 (13)	0.0230 (4)	
C30	0.1064 (3)	0.3543 (2)	−0.03732 (14)	0.0319 (5)	
H30A	0.1140	0.3318	−0.0889	0.048*	
H30B	0.1568	0.4128	−0.0383	0.048*	
H30C	0.0067	0.3877	−0.0282	0.048*	
C31	0.3219 (3)	0.1862 (3)	0.01054 (16)	0.0360 (6)	
H31A	0.3653	0.1166	0.0536	0.054*	
H31B	0.3782	0.2414	0.0050	0.054*	
H31C	0.3185	0.1626	−0.0402	0.054*	
C32	0.0853 (3)	0.1578 (2)	0.03751 (15)	0.0278 (5)	
H32A	−0.0135	0.1969	0.0454	0.042*	
H32B	0.1233	0.0909	0.0833	0.042*	
H32C	0.0905	0.1299	−0.0120	0.042*	
C33	0.0451 (2)	0.70618 (19)	0.27538 (13)	0.0203 (4)	
C34	0.0515 (2)	0.7391 (2)	0.34773 (13)	0.0217 (4)	
H34	0.0737	0.6807	0.3969	0.026*	
C35	0.0256 (2)	0.8566 (2)	0.34845 (14)	0.0226 (4)	
C36	−0.0068 (2)	0.9434 (2)	0.27575 (14)	0.0230 (4)	
C37	−0.0160 (2)	0.9096 (2)	0.20417 (14)	0.0238 (4)	
H37	−0.0399	0.9678	0.1550	0.029*	
C38	0.0092 (2)	0.7919 (2)	0.20356 (13)	0.0230 (4)	
H38	0.0019	0.7701	0.1544	0.028*	
C39	0.0356 (3)	0.8902 (2)	0.42721 (14)	0.0281 (5)	
H39A	0.0529	0.8201	0.4716	0.042*	
H39B	−0.0533	0.9476	0.4341	0.042*	
H39C	0.1135	0.9249	0.4270	0.042*	
C40	−0.0267 (3)	1.0705 (2)	0.27440 (15)	0.0279 (5)	
H40A	−0.1093	1.0992	0.3042	0.042*	
H40B	−0.0414	1.1169	0.2186	0.042*	
H40C	0.0575	1.0782	0.2995	0.042*	

### (E)-1-{2,2-Dibromo-1-[4-(tert-butyl)phenyl]ethenyl}-\ 2-(3,4-dimethylphenyl)diazene Atomic displacement parameters (Å^2^)

**Table:**

	*U*^11^	*U*^22^	*U*^33^	*U*^12^	*U*^13^	*U*^23^
Br1	0.04118 (15)	0.02158 (13)	0.01649 (12)	−0.00428 (10)	0.00179 (9)	−0.00884 (9)
Br2	0.04033 (14)	0.01623 (12)	0.02161 (12)	−0.00799 (9)	0.00190 (9)	−0.00576 (9)
N1	0.0230 (8)	0.0182 (8)	0.0171 (8)	−0.0051 (7)	−0.0006 (6)	−0.0062 (7)
N2	0.0222 (8)	0.0228 (9)	0.0183 (8)	−0.0068 (7)	−0.0011 (6)	−0.0056 (7)
C1	0.0225 (10)	0.0165 (9)	0.0177 (9)	−0.0035 (7)	−0.0009 (7)	−0.0066 (7)
C2	0.0279 (10)	0.0178 (10)	0.0188 (10)	−0.0057 (8)	0.0004 (8)	−0.0083 (8)
C3	0.0271 (10)	0.0158 (9)	0.0154 (9)	−0.0069 (8)	−0.0002 (7)	−0.0065 (7)
C4	0.0219 (10)	0.0219 (10)	0.0222 (10)	−0.0042 (8)	0.0011 (8)	−0.0105 (8)
C5	0.0234 (10)	0.0218 (10)	0.0210 (10)	−0.0095 (8)	0.0022 (8)	−0.0090 (8)
C6	0.0275 (10)	0.0178 (9)	0.0105 (8)	−0.0086 (8)	0.0029 (7)	−0.0060 (7)
C7	0.0206 (9)	0.0184 (10)	0.0204 (10)	−0.0041 (7)	0.0002 (7)	−0.0046 (8)
C8	0.0238 (10)	0.0200 (10)	0.0243 (10)	−0.0092 (8)	−0.0001 (8)	−0.0042 (8)
C9	0.0281 (10)	0.0153 (9)	0.0168 (9)	−0.0065 (8)	0.0013 (8)	−0.0041 (7)
C10	0.0379 (13)	0.0255 (11)	0.0283 (12)	−0.0130 (10)	0.0072 (10)	−0.0029 (9)
C11	0.0543 (16)	0.0242 (12)	0.0329 (13)	−0.0115 (11)	−0.0082 (11)	−0.0127 (10)
C12	0.0348 (12)	0.0180 (10)	0.0349 (13)	−0.0063 (9)	0.0047 (10)	−0.0065 (9)
C13	0.0197 (10)	0.0289 (11)	0.0184 (10)	−0.0064 (8)	−0.0009 (7)	−0.0078 (8)
C14	0.0246 (10)	0.0237 (10)	0.0215 (10)	−0.0066 (8)	−0.0006 (8)	−0.0048 (8)
C15	0.0212 (10)	0.0265 (11)	0.0213 (10)	−0.0055 (8)	0.0003 (8)	−0.0013 (8)
C16	0.0234 (10)	0.0279 (11)	0.0234 (11)	−0.0072 (8)	−0.0004 (8)	−0.0073 (9)
C17	0.0290 (11)	0.0234 (11)	0.0241 (11)	−0.0077 (9)	0.0019 (8)	−0.0089 (9)
C18	0.0249 (10)	0.0251 (11)	0.0214 (10)	−0.0063 (8)	−0.0005 (8)	−0.0074 (8)
C19	0.0415 (13)	0.0293 (12)	0.0204 (11)	−0.0097 (10)	0.0025 (9)	−0.0027 (9)
C20	0.0423 (13)	0.0351 (13)	0.0245 (11)	−0.0148 (11)	0.0055 (10)	−0.0125 (10)
Br3	0.04488 (15)	0.01919 (13)	0.02299 (13)	−0.00876 (10)	−0.00063 (10)	−0.00254 (9)
Br4	0.05341 (17)	0.02801 (14)	0.01713 (12)	−0.01054 (11)	−0.00191 (10)	−0.00619 (10)
N3	0.0276 (9)	0.0195 (9)	0.0194 (8)	−0.0081 (7)	0.0000 (7)	−0.0041 (7)
N4	0.0227 (8)	0.0203 (9)	0.0201 (8)	−0.0077 (7)	0.0003 (6)	−0.0066 (7)
C21	0.0249 (10)	0.0215 (10)	0.0190 (10)	−0.0080 (8)	0.0000 (8)	−0.0055 (8)
C22	0.0332 (11)	0.0212 (10)	0.0190 (10)	−0.0085 (9)	−0.0002 (8)	−0.0063 (8)
C23	0.0282 (10)	0.0171 (9)	0.0174 (9)	−0.0082 (8)	−0.0002 (8)	−0.0037 (8)
C24	0.0227 (10)	0.0237 (10)	0.0235 (10)	−0.0057 (8)	0.0018 (8)	−0.0073 (8)
C25	0.0230 (10)	0.0231 (10)	0.0222 (10)	−0.0060 (8)	−0.0027 (8)	−0.0063 (8)
C26	0.0241 (10)	0.0174 (9)	0.0156 (9)	−0.0060 (7)	−0.0001 (7)	−0.0019 (7)
C27	0.0221 (10)	0.0205 (10)	0.0205 (10)	−0.0058 (8)	0.0008 (8)	−0.0038 (8)
C28	0.0233 (10)	0.0211 (10)	0.0208 (10)	−0.0070 (8)	−0.0031 (8)	−0.0032 (8)
C29	0.0288 (11)	0.0231 (10)	0.0181 (10)	−0.0084 (8)	0.0001 (8)	−0.0059 (8)
C30	0.0501 (15)	0.0291 (12)	0.0187 (10)	−0.0178 (11)	−0.0060 (10)	−0.0016 (9)
C31	0.0335 (13)	0.0521 (16)	0.0266 (12)	−0.0105 (11)	0.0049 (10)	−0.0203 (12)
C32	0.0375 (12)	0.0253 (11)	0.0249 (11)	−0.0122 (9)	0.0007 (9)	−0.0103 (9)
C33	0.0211 (9)	0.0194 (10)	0.0211 (10)	−0.0066 (8)	0.0008 (7)	−0.0051 (8)
C34	0.0229 (10)	0.0230 (10)	0.0182 (10)	−0.0066 (8)	0.0009 (7)	−0.0033 (8)
C35	0.0208 (10)	0.0243 (11)	0.0230 (10)	−0.0047 (8)	0.0012 (8)	−0.0081 (9)
C36	0.0199 (10)	0.0210 (10)	0.0270 (11)	−0.0039 (8)	0.0006 (8)	−0.0063 (9)
C37	0.0264 (10)	0.0217 (10)	0.0212 (10)	−0.0073 (8)	−0.0027 (8)	−0.0005 (8)
C38	0.0257 (10)	0.0254 (11)	0.0185 (10)	−0.0083 (8)	−0.0018 (8)	−0.0051 (8)
C39	0.0366 (12)	0.0241 (11)	0.0232 (11)	−0.0043 (9)	0.0000 (9)	−0.0104 (9)
C40	0.0303 (11)	0.0209 (11)	0.0311 (12)	−0.0048 (9)	−0.0007 (9)	−0.0070 (9)

### (E)-1-{2,2-Dibromo-1-[4-(tert-butyl)phenyl]ethenyl}-\ 2-(3,4-dimethylphenyl)diazene Geometric parameters (Å, º)

**Table:**

Br1—C2	1.870 (2)	Br3—C22	1.881 (2)
Br2—C2	1.882 (2)	Br4—C22	1.876 (2)
N1—N2	1.262 (3)	N3—N4	1.260 (3)
N1—C1	1.415 (3)	N3—C21	1.413 (3)
N2—C13	1.424 (3)	N4—C33	1.431 (3)
C1—C2	1.346 (3)	C21—C22	1.349 (3)
C1—C3	1.493 (3)	C21—C23	1.487 (3)
C3—C8	1.387 (3)	C23—C28	1.389 (3)
C3—C4	1.391 (3)	C23—C24	1.398 (3)
C4—C5	1.388 (3)	C24—C25	1.382 (3)
C4—H4	0.9500	C24—H24	0.9500
C5—C6	1.401 (3)	C25—C26	1.403 (3)
C5—H5	0.9500	C25—H25	0.9500
C6—C7	1.390 (3)	C26—C27	1.393 (3)
C6—C9	1.533 (3)	C26—C29	1.532 (3)
C7—C8	1.397 (3)	C27—C28	1.392 (3)
C7—H7	0.9500	C27—H27	0.9500
C8—H8	0.9500	C28—H28	0.9500
C9—C11	1.527 (3)	C29—C31	1.525 (3)
C9—C10	1.533 (3)	C29—C32	1.537 (3)
C9—C12	1.533 (3)	C29—C30	1.540 (3)
C10—H10A	0.9800	C30—H30A	0.9800
C10—H10B	0.9800	C30—H30B	0.9800
C10—H10C	0.9800	C30—H30C	0.9800
C11—H11A	0.9800	C31—H31A	0.9800
C11—H11B	0.9800	C31—H31B	0.9800
C11—H11C	0.9800	C31—H31C	0.9800
C12—H12A	0.9800	C32—H32A	0.9800
C12—H12B	0.9800	C32—H32B	0.9800
C12—H12C	0.9800	C32—H32C	0.9800
C13—C14	1.388 (3)	C33—C38	1.392 (3)
C13—C18	1.406 (3)	C33—C34	1.396 (3)
C14—C15	1.405 (3)	C34—C35	1.389 (3)
C14—H14	0.9500	C34—H34	0.9500
C15—C16	1.398 (3)	C35—C36	1.408 (3)
C15—C19	1.508 (3)	C35—C39	1.511 (3)
C16—C17	1.393 (3)	C36—C37	1.393 (3)
C16—C20	1.507 (3)	C36—C40	1.505 (3)
C17—C18	1.385 (3)	C37—C38	1.391 (3)
C17—H17	0.9500	C37—H37	0.9500
C18—H18	0.9500	C38—H38	0.9500
C19—H19A	0.9800	C39—H39A	0.9800
C19—H19B	0.9800	C39—H39B	0.9800
C19—H19C	0.9800	C39—H39C	0.9800
C20—H20A	0.9800	C40—H40A	0.9800
C20—H20B	0.9800	C40—H40B	0.9800
C20—H20C	0.9800	C40—H40C	0.9800
			
N2—N1—C1	113.51 (17)	N4—N3—C21	115.01 (18)
N1—N2—C13	113.24 (18)	N3—N4—C33	112.37 (18)
C2—C1—N1	116.73 (18)	C22—C21—N3	113.99 (19)
C2—C1—C3	121.99 (18)	C22—C21—C23	123.0 (2)
N1—C1—C3	121.17 (18)	N3—C21—C23	122.99 (19)
C1—C2—Br1	122.95 (16)	C21—C22—Br4	123.41 (17)
C1—C2—Br2	123.01 (16)	C21—C22—Br3	122.78 (17)
Br1—C2—Br2	114.03 (11)	Br4—C22—Br3	113.81 (12)
C8—C3—C4	119.14 (19)	C28—C23—C24	118.8 (2)
C8—C3—C1	118.81 (18)	C28—C23—C21	120.03 (19)
C4—C3—C1	122.02 (19)	C24—C23—C21	121.2 (2)
C5—C4—C3	120.2 (2)	C25—C24—C23	120.2 (2)
C5—C4—H4	119.9	C25—C24—H24	119.9
C3—C4—H4	119.9	C23—C24—H24	119.9
C4—C5—C6	121.55 (19)	C24—C25—C26	121.8 (2)
C4—C5—H5	119.2	C24—C25—H25	119.1
C6—C5—H5	119.2	C26—C25—H25	119.1
C7—C6—C5	117.50 (19)	C27—C26—C25	117.30 (19)
C7—C6—C9	122.46 (19)	C27—C26—C29	123.09 (19)
C5—C6—C9	120.04 (18)	C25—C26—C29	119.61 (18)
C6—C7—C8	121.32 (19)	C28—C27—C26	121.3 (2)
C6—C7—H7	119.3	C28—C27—H27	119.3
C8—C7—H7	119.3	C26—C27—H27	119.3
C3—C8—C7	120.33 (19)	C23—C28—C27	120.64 (19)
C3—C8—H8	119.8	C23—C28—H28	119.7
C7—C8—H8	119.8	C27—C28—H28	119.7
C11—C9—C6	110.07 (18)	C31—C29—C26	112.15 (18)
C11—C9—C10	109.4 (2)	C31—C29—C32	108.6 (2)
C6—C9—C10	109.04 (18)	C26—C29—C32	109.08 (18)
C11—C9—C12	108.1 (2)	C31—C29—C30	108.6 (2)
C6—C9—C12	111.90 (18)	C26—C29—C30	108.85 (18)
C10—C9—C12	108.28 (19)	C32—C29—C30	109.58 (19)
C9—C10—H10A	109.5	C29—C30—H30A	109.5
C9—C10—H10B	109.5	C29—C30—H30B	109.5
H10A—C10—H10B	109.5	H30A—C30—H30B	109.5
C9—C10—H10C	109.5	C29—C30—H30C	109.5
H10A—C10—H10C	109.5	H30A—C30—H30C	109.5
H10B—C10—H10C	109.5	H30B—C30—H30C	109.5
C9—C11—H11A	109.5	C29—C31—H31A	109.5
C9—C11—H11B	109.5	C29—C31—H31B	109.5
H11A—C11—H11B	109.5	H31A—C31—H31B	109.5
C9—C11—H11C	109.5	C29—C31—H31C	109.5
H11A—C11—H11C	109.5	H31A—C31—H31C	109.5
H11B—C11—H11C	109.5	H31B—C31—H31C	109.5
C9—C12—H12A	109.5	C29—C32—H32A	109.5
C9—C12—H12B	109.5	C29—C32—H32B	109.5
H12A—C12—H12B	109.5	H32A—C32—H32B	109.5
C9—C12—H12C	109.5	C29—C32—H32C	109.5
H12A—C12—H12C	109.5	H32A—C32—H32C	109.5
H12B—C12—H12C	109.5	H32B—C32—H32C	109.5
C14—C13—C18	119.1 (2)	C38—C33—C34	119.8 (2)
C14—C13—N2	116.2 (2)	C38—C33—N4	115.74 (19)
C18—C13—N2	124.7 (2)	C34—C33—N4	124.42 (19)
C13—C14—C15	121.8 (2)	C35—C34—C33	120.5 (2)
C13—C14—H14	119.1	C35—C34—H34	119.7
C15—C14—H14	119.1	C33—C34—H34	119.7
C16—C15—C14	118.5 (2)	C34—C35—C36	119.9 (2)
C16—C15—C19	121.8 (2)	C34—C35—C39	119.7 (2)
C14—C15—C19	119.7 (2)	C36—C35—C39	120.4 (2)
C17—C16—C15	119.7 (2)	C37—C36—C35	119.0 (2)
C17—C16—C20	120.0 (2)	C37—C36—C40	120.1 (2)
C15—C16—C20	120.3 (2)	C35—C36—C40	120.9 (2)
C18—C17—C16	121.6 (2)	C38—C37—C36	121.1 (2)
C18—C17—H17	119.2	C38—C37—H37	119.5
C16—C17—H17	119.2	C36—C37—H37	119.5
C17—C18—C13	119.2 (2)	C37—C38—C33	119.7 (2)
C17—C18—H18	120.4	C37—C38—H38	120.2
C13—C18—H18	120.4	C33—C38—H38	120.2
C15—C19—H19A	109.5	C35—C39—H39A	109.5
C15—C19—H19B	109.5	C35—C39—H39B	109.5
H19A—C19—H19B	109.5	H39A—C39—H39B	109.5
C15—C19—H19C	109.5	C35—C39—H39C	109.5
H19A—C19—H19C	109.5	H39A—C39—H39C	109.5
H19B—C19—H19C	109.5	H39B—C39—H39C	109.5
C16—C20—H20A	109.5	C36—C40—H40A	109.5
C16—C20—H20B	109.5	C36—C40—H40B	109.5
H20A—C20—H20B	109.5	H40A—C40—H40B	109.5
C16—C20—H20C	109.5	C36—C40—H40C	109.5
H20A—C20—H20C	109.5	H40A—C40—H40C	109.5
H20B—C20—H20C	109.5	H40B—C40—H40C	109.5
			
C1—N1—N2—C13	178.45 (17)	C21—N3—N4—C33	−176.50 (17)
N2—N1—C1—C2	177.35 (19)	N4—N3—C21—C22	177.8 (2)
N2—N1—C1—C3	−6.3 (3)	N4—N3—C21—C23	−0.2 (3)
N1—C1—C2—Br1	179.63 (14)	N3—C21—C22—Br4	−2.6 (3)
C3—C1—C2—Br1	3.3 (3)	C23—C21—C22—Br4	175.41 (16)
N1—C1—C2—Br2	1.0 (3)	N3—C21—C22—Br3	178.31 (15)
C3—C1—C2—Br2	−175.32 (15)	C23—C21—C22—Br3	−3.7 (3)
C2—C1—C3—C8	89.0 (3)	C22—C21—C23—C28	−64.6 (3)
N1—C1—C3—C8	−87.1 (3)	N3—C21—C23—C28	113.2 (2)
C2—C1—C3—C4	−93.1 (3)	C22—C21—C23—C24	115.3 (3)
N1—C1—C3—C4	90.7 (3)	N3—C21—C23—C24	−66.9 (3)
C8—C3—C4—C5	0.0 (3)	C28—C23—C24—C25	0.6 (3)
C1—C3—C4—C5	−177.83 (19)	C21—C23—C24—C25	−179.4 (2)
C3—C4—C5—C6	−0.6 (3)	C23—C24—C25—C26	−0.5 (3)
C4—C5—C6—C7	0.6 (3)	C24—C25—C26—C27	−0.1 (3)
C4—C5—C6—C9	−179.88 (19)	C24—C25—C26—C29	−179.4 (2)
C5—C6—C7—C8	0.0 (3)	C25—C26—C27—C28	0.7 (3)
C9—C6—C7—C8	−179.51 (19)	C29—C26—C27—C28	179.9 (2)
C4—C3—C8—C7	0.6 (3)	C24—C23—C28—C27	0.0 (3)
C1—C3—C8—C7	178.49 (19)	C21—C23—C28—C27	180.0 (2)
C6—C7—C8—C3	−0.6 (3)	C26—C27—C28—C23	−0.7 (3)
C7—C6—C9—C11	−124.5 (2)	C27—C26—C29—C31	0.0 (3)
C5—C6—C9—C11	56.0 (3)	C25—C26—C29—C31	179.2 (2)
C7—C6—C9—C10	115.5 (2)	C27—C26—C29—C32	120.3 (2)
C5—C6—C9—C10	−64.0 (2)	C25—C26—C29—C32	−60.5 (3)
C7—C6—C9—C12	−4.3 (3)	C27—C26—C29—C30	−120.2 (2)
C5—C6—C9—C12	176.27 (19)	C25—C26—C29—C30	59.0 (3)
N1—N2—C13—C14	−179.04 (18)	N3—N4—C33—C38	179.90 (19)
N1—N2—C13—C18	0.2 (3)	N3—N4—C33—C34	1.9 (3)
C18—C13—C14—C15	−0.7 (3)	C38—C33—C34—C35	−1.6 (3)
N2—C13—C14—C15	178.53 (19)	N4—C33—C34—C35	176.37 (19)
C13—C14—C15—C16	−1.0 (3)	C33—C34—C35—C36	−0.2 (3)
C13—C14—C15—C19	179.2 (2)	C33—C34—C35—C39	−178.9 (2)
C14—C15—C16—C17	2.3 (3)	C34—C35—C36—C37	1.7 (3)
C19—C15—C16—C17	−177.9 (2)	C39—C35—C36—C37	−179.6 (2)
C14—C15—C16—C20	−177.3 (2)	C34—C35—C36—C40	−176.3 (2)
C19—C15—C16—C20	2.5 (3)	C39—C35—C36—C40	2.4 (3)
C15—C16—C17—C18	−1.9 (3)	C35—C36—C37—C38	−1.3 (3)
C20—C16—C17—C18	177.7 (2)	C40—C36—C37—C38	176.7 (2)
C16—C17—C18—C13	0.1 (3)	C36—C37—C38—C33	−0.4 (3)
C14—C13—C18—C17	1.2 (3)	C34—C33—C38—C37	1.9 (3)
N2—C13—C18—C17	−178.0 (2)	N4—C33—C38—C37	−176.22 (19)

## References

[bb1] Akkurt, M., Yıldırım, S. Ö., Shikhaliyev, N. Q., Mammadova, N. A., Niyazova, A. A., Khrustalev, V. N. & Bhattarai, A. (2022). *Acta Cryst.* E**78**, 732–736. 10.1107/S205698902200620X PMC9260355 35855357

[bb2] Allen, F. H., Kennard, O., Watson, D. G., Brammer, L., Orpen, A. G. & Taylor, R. (1987). *J. Chem. Soc. Perkin Trans. 2* pp. S1–S19.

[bb3] Atioğlu, Z., Akkurt, M., Shikhaliyev, N. Q., Mammadova, N. A., Babayeva, G. V., Khrustalev, V. N. & Bhattarai, A. (2022*a*). *Acta Cryst.* E**78**, 530–535. 10.1107/S2056989022004388 PMC9069519 35547788

[bb4] Atioğlu, Z., Akkurt, M., Shikhaliyev, N. Q., Mammadova, N. A., Babayeva, G. V., Khrustalev, V. N. & Bhattarai, A. (2022*b*). *Acta Cryst.* E**78**, 804–808. 10.1107/S2056989022007113 PMC9361372 35974833

[bb9] Carter-Fenk, K. & Herbert, J. M. (2020). *Phys. Chem. Chem. Phys.* **22**, 24870–24886. 10.1039/d0cp05039c 33107520

[bb7] Benkhaya, S., M’rabet, S. & El Harfi, A. (2020). *Heliyon*, **6**, e03271. 10.1016/j.heliyon.2020.e03271 PMC7002841 32042981

[bb10] Cavallo, G., Metrangolo, P., Milani, R., Pilati, T., Priimagi, A., Resnati, G. & Terraneo, G. (2016). *Chem. Rev.* **116**, 2478–2601. 10.1021/acs.chemrev.5b00484 PMC4768247 26812185

[bb11] Çelikesir, S. T., Akkurt, M., Shikhaliyev, N. Q., Mammadova, N. A., Suleymanova, G. T., Khrustalev, V. N. & Bhattarai, A. (2022). *Acta Cryst.* E**78**, 404–408. 10.1107/S205698902200278X PMC8983971 35492284

[bb12] Dobrounig, P., Trobe, M. & Breinbauer, R. (2017). *Monatsh. Chem.* **148**, 3–35. 10.1007/s00706-016-1883-7 PMC5225241 28127089

[bb13] Groom, C. R., Bruno, I. J., Lightfoot, M. P. & Ward, S. C. (2016). *Acta Cryst.* B**72**, 171–179. 10.1107/S2052520616003954 PMC4822653 27048719

[bb14] Gurbanov, A. V., Mertsalov, D. F., Zubkov, F. I., Nadirova, M. A., Nikitina, E. V., Truong, H. H., Grigoriev, M. S., Zaytsev, V. P., Mahmudov, K. T. & Pombeiro, A. J. L. (2021). *Crystals* **11**, 1–10.

[bb15] Israyilova, A., Buroni, S., Forneris, F., Scoffone, V. C., Shixaliyev, N. Q., Riccardi, G. & Chiarelli, L. R. (2016). *PLoS One* **11**, e0167350. 10.1371/journal.pone.0167350 PMC5127577 27898711

[bb17] Khalilov, A. N., Khrustalev, V. N., Tereshina, T. A., Akkurt, M., Rzayev, R. M., Akobirshoeva, A. A. & Mamedov, İ. G. (2022). *Acta Cryst.* E**78**, 525–529. 10.1107/S2056989022004297 PMC9069515 35547793

[bb18] Magerramov, A. M., Naghiyev, F. N., Mamedova, G. Z., Asadov, K. A. & Mamedov, I. G. (2018). *Russ. J. Org. Chem.* **54**, 1731–1734.

[bb19] Maharramov, A. M., Duruskari, G. S., Mammadova, G. Z., Khalilov, A. N., Aslanova, J. M., Cisterna, J., Cárdenas, A. & Brito, I. (2019). *J. Chil. Chem. Soc.* **64**, 4441–4447.

[bb20] Mahmoudi, G., Zangrando, E., Miroslaw, B., Gurbanov, A. V., Babashkina, M. G., Frontera, A. & Safin, D. A. (2021). *Inorg. Chim. Acta* **519**, 120279, 1–10.

[bb21] Marek, P. H., Urban, M. & Madura, I. D. (2018). *Acta Cryst.* C**74**, 1509–1517. 10.1107/S2053229618013608 30398208

[bb22] Nagarajan, K., Rajagopal, S. K. & Hariharan, M. (2014). *CrystEngComm* **16**, 8946–8949.

[bb23] Naghiyev, F., Mamedov, I., Khrustalev, V., Shixaliyev, N. & Maharramov, A. (2019). *J. Chin. Chem. Soc.* **66**, 253–256.

[bb24] Özkaraca, K., Akkurt, M., Shikhaliyev, N. Q., Askerova, U. F., Suleymanova, G. T., Mammadova, G. Z. & Shadrack, D. M. (2020*b*). *Acta Cryst.* E**76**, 1251–1254. 10.1107/S2056989020009202 PMC7405560 32844008

[bb25] Özkaraca, K., Akkurt, M., Shikhaliyev, N. Q., Askerova, U. F., Suleymanova, G. T., Shikhaliyeva, I. M. & Bhattarai, A. (2020*a*). *Acta Cryst.* E**76**, 811–815. 10.1107/S2056989020006106 PMC7274008 32523745

[bb26] Rajagopal, S. K., Salini, P. S. & Hariharan, M. (2016). *Cryst. Growth Des.* **16**, 4567–4573.

[bb43] Rigaku OD (2021). *CrysAlis PRO.* Rigaku Oxford Diffraction, Yarnton, England.

[bb27] Sheldrick, G. M. (2008). *Acta Cryst.* A**64**, 112–122. 10.1107/S0108767307043930 18156677

[bb28] Sheldrick, G. M. (2015*a*). *Acta Cryst.* A**71**, 3–8.

[bb29] Sheldrick, G. M. (2015*b*). *Acta Cryst.* C**71**, 3–8.

[bb30] Shikhaliyev, N. G., Maharramov, A. M., Suleymanova, G. T., Babazade, A. A., Nenajdenko, V. G., Khrustalev, V. N., Novikov, A. S. & Tskhovrebov, A. G. (2021). *Mendeleev Commun.* **31**, 677–679.

[bb31] Shikhaliyev, N. G., Suleymanova, G. T., İsrayilova, A. A., Ganbarov, K. G., Babayeva, G. V., Garazadeh, K. A., Mammadova, G. Z. & Nenajdenko, V. G. (2019). *Arkivoc* **2019**, 64–73.

[bb32] Shikhaliyev, N. Q., Ahmadova, N. E., Gurbanov, A. V., Maharramov, A. M., Mammadova, G. Z., Nenajdenko, V. G., Zubkov, F. I., Mahmudov, K. T. & Pombeiro, A. J. L. (2018). *Dyes Pigments* **150**, 377–381.

[bb33] Shixaliyev, N. Q., Maharramov, A. M., Gurbanov, A. V., Gurbanova, N. V., Nenajdenko, V. G., Muzalevskiy, V. M., Mahmudov, K. T. & Kopylovich, M. N. (2013). *J. Mol. Struct.* **1041**, 213–218.

[bb39] Steiner, T. (2002). *Angew. Chem. Int. Ed.* **41**, 48–76.

[bb40] Tahirli, S., Sadeghian, N., Aliyeva, F., Sujayev, A., Günay, S., Erden, Y., Shikhaliyev, N., Kaya, S., Mehtap Özden, E., Chiragov, F., Berisha, A. & Taslimi, P. (2024). *Chem. Biodivers.* **21**, e202301861. 10.1002/cbdv.202301861 38367267

[bb42] Varadwaj, P. R., Varadwaj, A. & Marques, H. M. (2019). *Inorganics* **7**, 40, 1–63.

